# A meta-analysis of the clinicopathological significance of the lncRNA MALAT1 in human gastric cancer

**DOI:** 10.3389/fonc.2023.1257120

**Published:** 2024-01-04

**Authors:** Shaoxiong Bai, Jiansheng Guo, Haofan Zhang

**Affiliations:** Gastrointestinal Surgery, The First Hospital of Shanxi Medical University, Taiyuan, Shanxi, China

**Keywords:** meta-analysis, lncRNA, MALAT1, gastric cancer, prognosis

## Abstract

**Background:**

Dysregulation of the long non-coding RNA metastasis-associated lung adenocarcinoma transcript 1 (MALAT1) has been linked to some oncogenic pathways that induce cancer initiation and progression. This meta-analysis was conducted to specifically summarize the most recent research on MALAT1 function in human gastric cancer (GC).

**Methods:**

The eligible studies were first identified by searching HowNet, Web of Science, PubMed, The Cochrane Library, Embase, and *Nature* databases for studies published as of April 1, 2023. The meta-analysis included 14 studies assessing MALAT1 expression and presenting clinical parameters and survival outcomes.

**Results:**

The results illustrated that high MALAT1 expression is predictive of lymph node metastasis (pooled odds ratio [OR] = 2.99, 95% confidence interval [CI] = 1.97–4.54, P < 0.001) and distant metastasis in GC (OR = 3.11, 95% CI = 1.68–5.75, P < 0.001). In addition, MALAT1 was associated with GC tumor invasion (T_3_/T_4_ vs. T_1_/T_2_: OR = 2.90, 95% CI = 1.90- 4.41, P <0.001) and TNM stage (III/IV vs I/II: OR = 2.93, 95% CI: 1.80-4.77, P <0.001). Additionally, higher MALAT-1 expression predicted poorer overall survival in patients with GC (hazard ratio = 1.64, 95% CI = 1.20–2.09, P < 0.001).

**Conclusions:**

The current findings suggest that the high MALAT1 expression is an adverse biomarker for prognostic outcomes, lymph node metastasis, TNM stage, and distant metastasis in GC and MALAT1 could be a prognostic biomarker for GC.

## 1 Introduction

Gastric cancer (GC) is the second most common cause of cancer-related death globally (1). Although the incidence of GC has declined in most regions, its impact on the global disease burden and public health persists (2). Number of deaths caused by GC in 2020, both sexes, all ages, is 768793, about 7.7% of all cancers (Source: Globocan 2020). GC is a heterogeneous disease with a complex pathogenesis and geographic variation. The prognosis of GC is difficult to predict (3, 4). Further research on various aspects of GC is required to better identify the pathogenesis of the disease and uncover more practical biomarkers that predict prognosis and response to therapy.

Long non-coding RNAs (lncRNAs) are functional RNA molecules with a length of more than 200 nucleotides. Most of them have a 5′ cap and 3′ poly-A tails. LncRNAs exert their biological roles by binding to DNA, RNA, or proteins. They can act as intracellular competitive endogenous RNAs to regulate gene expression by directly interacting with miRNAs. They can also modulate target gene expression by altering the binding of transcription factors to promoters. In addition, they can facilitate target genes’ activation or silencing by forming scaffolding complexes with effector molecules (5, 6). lncRNAs were revealed to be closely associated with changes in oncogenic phenotypes including cell proliferation, cell differentiation, invasion, apoptosis, and metastasis (7–10). Based on the published evidence, cancer-associated lncRNAs might represent candidate biomarkers for providing precise diagnoses, assessing individualized prognosis, evaluating targeted therapy, and predicting tumor differentiation (11–13).

The lncRNA metastasis-associated lung adenocarcinoma transcript 1 (MALAT1), also known as nuclear transcript 2, which has been mapped to human chromosome 11q13, is more than 8000 nucleotides in length (14, 15). Unlike other lncRNAs, MALAT1 is evolutionarily conserved and widely expressed. MALAT1 was originally identified as a metastasis-associated gene and prognostic marker that can be used to identify patients with metastatic early-stage non-small cell lung cancer (NSCLC) who are at high risk of developing metastatic exacerbation (16).

MALAT1 plays significant roles in numerous human cancers, such as regulating pre-messenger RNA splicing, transcription factors, and histone-modifying enzymes (17). Knockdown of MALAT1 in animal experiments inhibited cell migration and proliferation and resulted in reduced expression of genes such as enhancer of zeste homolog 2 (EZH2), Lin28, β-catenin, octamer-binding transcription factor 4 (OCT4), and epithelial–mesenchymal transition (EMT) (18). MALAT1 silencing might inversely regulate miR-129-5p to block triple-negative breast cancer cell migration, proliferation, and invasion (19). As a competing endogenous RNA (ceRNA), MALAT1 regulates Zinc finger E-box-binding homeobox 1 (ZEB1) expression by sponging miR-143-3p. Hepatocellular carcinoma cell proliferation and invasion can be inhibited by MALAT1 inhibition (20). Oncogenic phenotypic transition caused by MALAT1 was detected in GC (21). The *MALAT1* gene is overexpressed in GC and related to its occurrence and growth (22). By serving as a ceRNA for miR-23b-3p in chemotherapy-resistant GC cells, MALAT1 silencing was demonstrated to decrease chemotherapy-induced autophagy (23). In conclusion, accumulated data indicate a connection between MATAT1 dysregulation and the emergence of GC.

Over the past decade, increasing numbers of studies have proved the effect of MALAT1 expression on the prognostic outcomes and clinicopathological parameters of GC. Nevertheless, these studies produced inconsistent or controversial conclusions (22, 23). To resolve this issue, we performed a systematic review and meta-analysis to clarify the association of MALAT1 with GC prognostic or clinical features and recapitulate its tumorigenicity in GC.

## 2 Materials and methods

### 2.1 Document retrieval

We searched HowNet, Web of Science, PubMed, The Cochrane Library, Embase, and *Nature* databases for studies published through April 1, 2023. The following keyword combinations were used in this search: (“long noncoding RNA MALAT1” or “lncRNA MALAT1” or “MALAT1”) and (“gastric cancer” or “stomach tumor”). In addition, the reference lists of all qualified studies were retrieved to ensure that all eligible studies were included in the meta-analysis.

### 2.2 Study selection

The eligibility criteria were as follows: (1) randomized controlled trials (RCTs) or observational tests; (2) examination of the expression of MALAT1 in patients with GC; (3) separation of patients into high- and low-expression groups along with the expression level of MALAT1; (4) inclusion of overall survival (OS), recurrence-free survival (RFS), disease-free survival (DFS), or other clinical parameters with hazard ratios (HRs) and 95% confidence intervals (CIs) or data that can be used to calculate DFS or OS; and (5) the availability of sufficient data. The following studies were excluded from this study: (1) case reports, reviews, meta-analyses, letters, conference presentations, and editorials; (2) duplicated publications; (3) studies with animal experiments or pure cell experiments; and (4) papers with insufficient data.

### 2.3 Data extraction and quality assessment

Two authors independently completed data extraction and quality assessment. Any disagreements in the process were resolved by discussion with the third author. The following information was extracted from every article included in this study: name of the first author, year of publication, sex of patients, detection method, number of patients, and source (eg, tissue, serum) of lncRNA MALAT1, indicators in the article (eg, OS, DFS, RFS), and clinical parameters (eg, tumor size, TNM stage, metastasis). In terms of prognostic parameters, HRs and their corresponding 95% CIs were extracted from the article. If an article failed to report the HR or its 95% CI, this information was obtained indirectly, such as by extracting data from survival curves. In addition, the Newcastle–Ottawa Scale (NOS) was used to assess the quality of the included studies. First, some HRs were obtained directly from the article. Second, HRs were estimated using the formula [P0/(1 − P0)]/[P1/(1 − P1)], where P0 is the 5-year survival rate in the low MALAT1 expression group and P1 is the 5-year survival rate in the high MALAT1 expression group. The 95% CI was calculated using the formula exp(lnHR ± 1.96 × SE), where exp is the exponential function, lnHR is the natural logarithm of HR, and SE is the standard error of the HR. Finally, the data were obtained using the survival curves. If these data were analyzed by both univariate and multivariate methods, multivariate analysis was preferred because it has greater precision in explaining confounders. Study quality was assessed using NOS. NOS scores ranged from 0 to 9, and scores ≥ 6 denoted high study quality.

### 2.4 Statistical analysis

All analyses were performed using Stata 16.0. For prognostic values, such as OS, HRs and the corresponding 95% CIs were used to detect the overall effect. For clinical parameters such as TNM stage, lymph node metastasis, and tumor differentiation, odds ratios (ORs) and their corresponding 95% CIs were used. The *I^2^* test was used to assess the heterogeneity across studies. *I^2^* ≤ 50% indicated the absence of significant heterogeneity among these included studies, and the fixed-effects model was used in the analysis. Otherwise, the random-effects model was used. Funnel plots were made to distinguish bias among included studies. Publication bias in OS meta-analyses was also assessed by Egger’s test.

## 3 Results

### 3.1 Identification of the included studies

**Figure 1:**
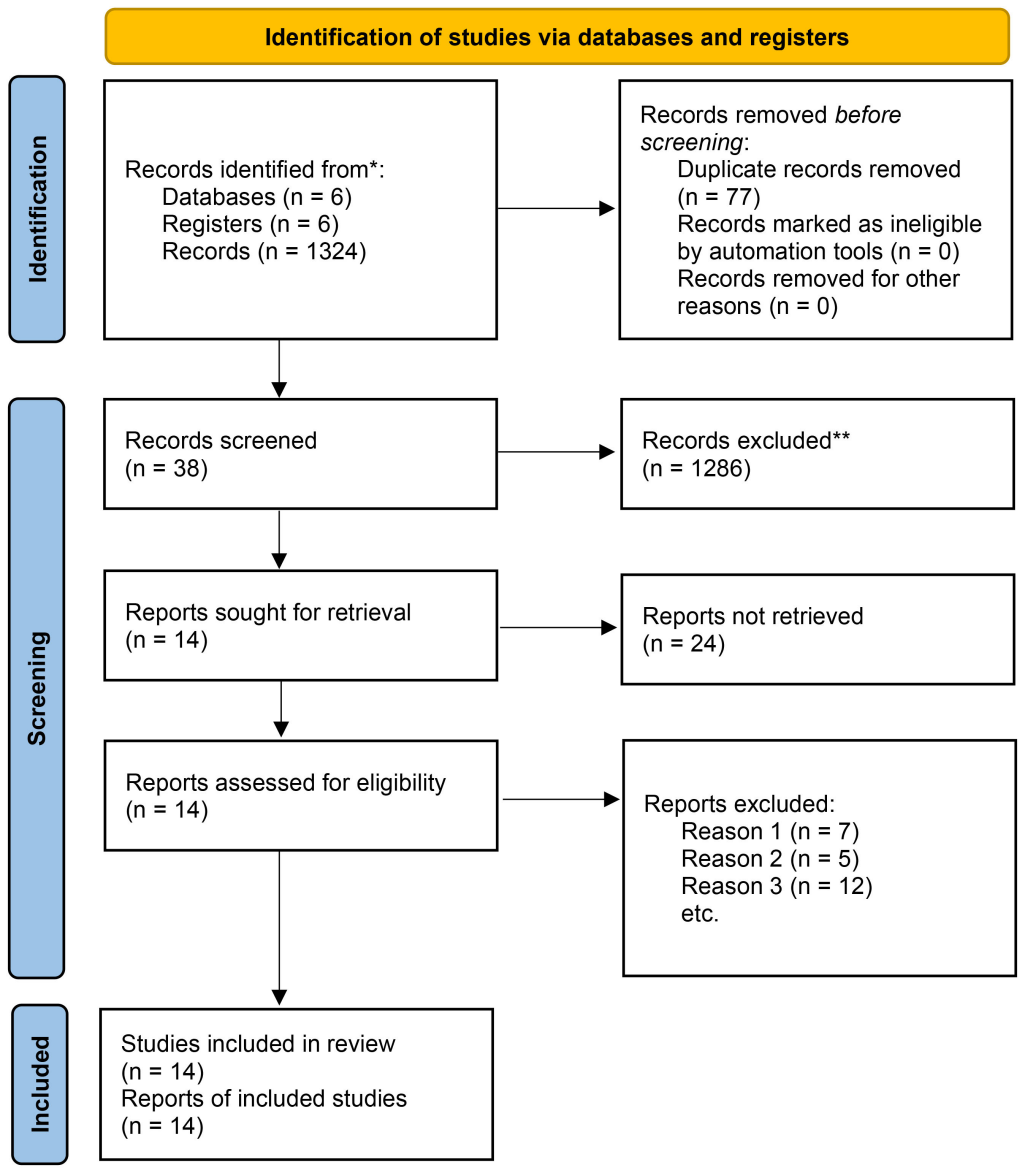
Flowchart of the articles and study selection process. *Consider, if feasible to do so, reporting the number of records identified from each database or register searched (rather than the total number across all databases/registers). **If automation tools were used, indicate how many records were excluded by a human and how many were excluded by automation tools.

As presented in **Figure 1**, 1401 studies were primarily retrieved. After excluding 77 duplicate publications, 1324 papers were retained. Subsequently, 1286 papers were directly excluded after reading the title or abstract, including 466 reviews, 632 irrelevant articles, 188 letters or books, and cell or animal experiments articles. Hence, the full text of 38 papers was reviewed, after which 24 papers were excluded, mainly because of incomplete data or incompatible topics. Ultimately, 14 studies were included in this study.

### 3.2 General information

The main characteristics of the included studies are presented in **Table 1**. The 14 included studies included 10, 2, 1, and 1 study from China, Iran, South Korea, and the United States, respectively, and the studies were published between 2014 and 2023. MALAT1 expression was determined in all studies by quantitative reverse transcription-polymerase chain reaction (qRT-PCR). The effect of high/low MALAT1 expression on stage, histologic grade, lymph node metastasis, distant metastasis, survival, and T stage were also analyzed. All studies included the comparison of MALAT1 expression between patients with GC and healthy controls, and MALAT1 expression was higher in patients with GC in all studies. Five studies reported OS data. Of these, three studies presented HRs and 95% CIs, and the needed data were calculated using Kaplan–Meier survival curves for two studies. The NOS score was ≥6 in all studies, indicating high study quality.

**Table 1:** Characteristics of the selected studies.

Author	Year	Country	Cases	Age (Case: Control)	Sex (female/male)	TNM stage	Lymph node metastasis (Y: N)	Distant metastasis (Y: N)	Studies (n)	Test material	Test method	Index	NOS score
Quan-Jun Deng (24)	2016	China	GC participants	NA	NA	NA	NA	NA	25	Gastric adenocarcinoma tissue and matched normal adjacent tissue	qRT-PCR	①	6
Yue Li (25)	2017	China	GC participants	<55 (64): ≥56 (86)	94:56	I16: II30: III54: IV50	118:32	50:100	150	GC tissue	qRT-PCR	①②③④⑤⑥⑦	6
Zhengmao Lu (26)	2019	China	GC participants	50.26 ± 12.02: 46.53 ± 11.14	39/31(Cases): 37/33(Controls)	NA	NA	NA	70:70	Serum	qRT-PCR	①	6
Jijun Li (27)	2017	China	GC participants	≤50 (45): > 50 (33)	42/36	I+II36: III+IV42	35:43	28: 50	78	Gastric adenocarcinoma tissue and matched normal adjacent tissue	qRT-PCR	①②③④⑤⑥⑦	7
Na Keum Lee (28)	2017	South Korea	GC participants	<65 (24): ≥65 (23)	32/15	I+II21: III 26	31:16	NA	50	Gastric adenocarcinoma tissue and matched normal adjacent tissue	qRT-PCR	①②④⑦	6
Yoshinaga Okugawa (21)	2014	USA	GC participants	<69 (71): ≥69 (79)	119/31	NA	105:45	27: 123	150	GC tissue and corresponding noncancerous gastric mucosa	qRT-PCR	①④⑥⑦	6
Kongxi Zhu (29)	2019	China	GC participants	42 ± 13.1	30/34	NA	NA	37: 27	64	Blood sample	RT-qPCR	①⑤⑥	8
Guoyi Shao (30)	2020	China	GC participants	56.3 (37-72)	39/18	NA	NA	NA	57	GC tissue and corresponding noncancerous adjacent tissue	RT-qPCR	①	7
Di Chen (31)	2017	China	GC participants	NA	NA	NA	NA	NA	20	GC and adjacent normal mucosa	qRT-PCR	①	6
Hongwei Xia (32)	2016	China	GC participants	NA	NA	NA	NA	25: 14	39	Gastric adenocarcinoma tissue and matched normal adjacent tissue	Q-PCR	①⑥	7
Vahid Chaleshi (33)	2021	Iran	GC participants	60.32 ± 14.185	34/7	I4: III22: IV15	4:34	NA	41	Gastric adenocarcinoma tissue and matched normal adjacent tissue	qRT-PCR	①	7
Yue Zhang (34)	2017	China	GC participants	≤60 (40): > 60 (20)	38/22		39:21	NA	60	GC tissue and adjacent normal tissue	qRT-PCR	①②③④⑦	7
Farbod Esfandi (35)	2020	Iran	GC participants	42.53 (14–55)	22/6	I1: II9: III13: IV6	24:5	24: 5	30	Gastric cancer tissue and adjacent normal tissue	qRT-PCR	①	7
Xiaoning Li (36)	2022	China	GC participants	59 ± 10	24/13	I12: II4: III14: IV7	28:9	4: 33	37	GC tissue and adjacent normal tissue	qRT-PCR	①	7

① Expression level. ② The effect of high/low MALAT1 expression on stage. ③ The effect of high/low MALAT1 expression on the histologic grade. ④ The effect of high/low MALAT1 expression on lymph node metastasis. ⑤ The effect of high/low MALAT1 expression on distant metastasis. ⑥ The effect of high/low MALAT1 expression on survival. ⑦ The effect of high/low MALAT1 expression on the T stage. NA, Not Available.

### 3.3 Meta-analysis of clinical parameters

**Figure 2:**
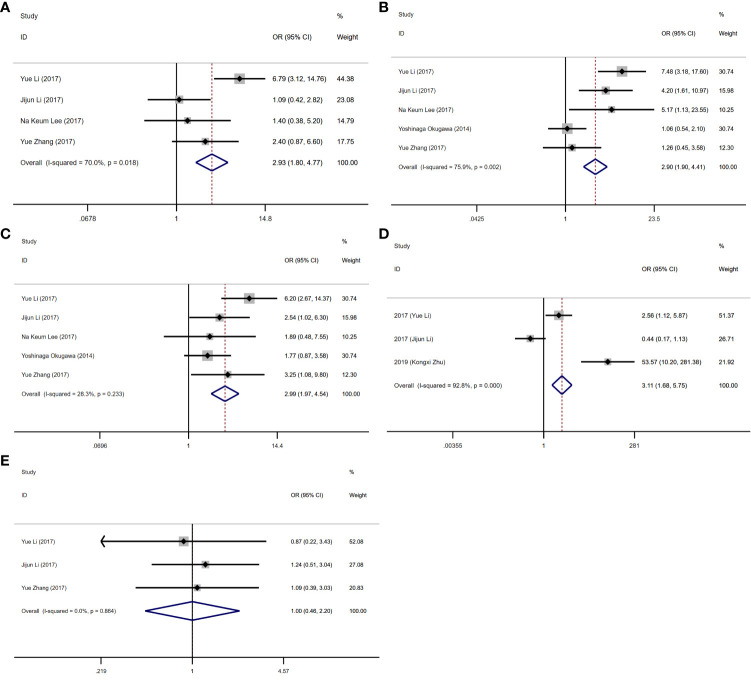
Forest plots of clinicopathological parameters. **(A)** Forest plot of TNM stage. **(B)** Forest plot of tumor invasion. **(C)** Forest plot of lymph node metastasis. **(D)** Forest plot of distant metastasis. **(E)** Forest plot of the histologic grade.

**Figure 3:**
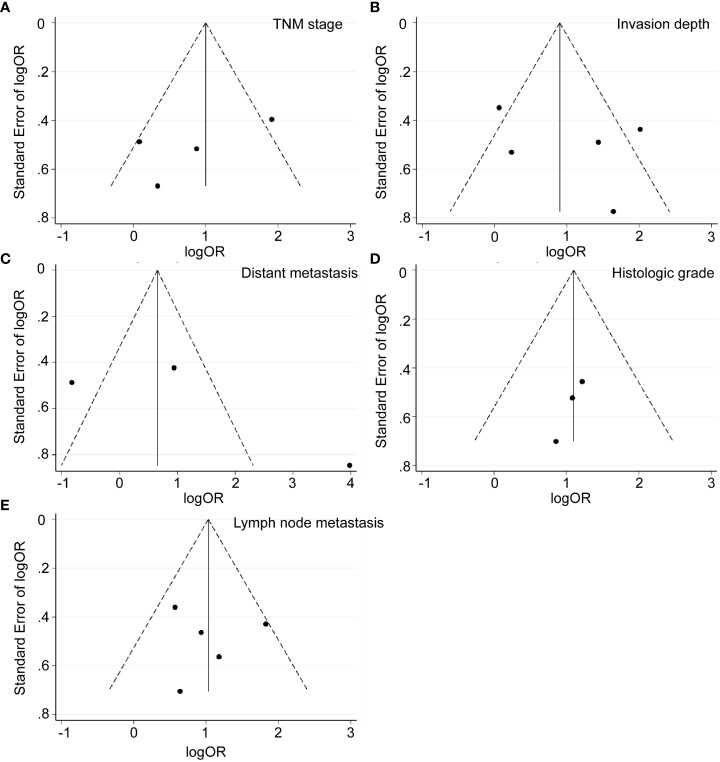
Funnel plots of clinicopathological parameters. **(A)** TNM stage (stage III/IV vs. Stage I/II). **(B)** Invasion depth (T3/T4 vs. T1/T2). **(C)** Distant metastasis (positive vs. negative). **(D)** Histologic grade (poorly differentiated vs. well-differentiated). **(E)** Lymph node metastasis (positive vs. negative).

As presented in **Table 2** and **Figures 2**, **3**, seven studies were included in the meta-analysis of clinical parameters. Four studies reported the TNM stage (**Figure 2A**), and high MALAT1 expression predicted an advanced TNM stage (III/IV vs. I/II: OR = 2.93, 95% CI = 1.80–4.77, P < 0.001). Meanwhile, heterogeneity was high among the studies (*I^2^ = *70.00%, P_Q_ = 0.018). Five studies included the stage of tumor invasion (**Figure 2B**), and high MALAT1 expression was significantly associated with tumor invasion (T_3_/T_4_ vs. T_1_/T_2_ OR = 2.90, 95% CI = 1.90–4.41, P < 0.001). Heterogeneity was high among the studies (I^2^ = 74.90%, P_Q_ = 0.002). Five studies included data on lymph node metastasis (**Figure 2C**), and high MALAT1 expression was significantly related to the presence of lymph node metastasis (positive vs. negative: pooled OR = 2.99, 95% CI = 1.97–4.54, P < 0.001). No significant heterogeneity was detected among the studies (*I^2^ = *28.30%, P_Q_ = 0.788). In addition, three studies reported data on distant metastasis (**Figure 2D**), and high MALAT1 expression was significantly associated with distant metastasis (positive vs. negative: OR = 3.11, 95% CI = 1.68–5.75, P < 0.001). Significant heterogeneity was detected across the studies (*I^2^ = *92.8%, P_Q_ < 0.001). Three studies reported the tumor histologic grade (**Figure 2E**). High MALAT1 expression was not associated with the tumor histologic grade (poorly differentiated vs. well-differentiated: OR = 1.00, 95% CI = 0.46–2.20, P = 0.997), and no significant heterogeneity was noted across the studies (*I^2^ = *0.0%, P_Q_ = 0.864). Egger’s test was performed to assess publication bias in the studies, and no significant publication bias was detected (P > 0.05). The symmetrical funnel plot revealed symmetry among clinicopathological outcomes (**Figure 3**).

**Table 2:** The clinical parameter META analysis.

Clinicopathological parameters	Studies (n)	Patients (n)	OR (95%CI)	*P*	*I^2^*	*P_Q_*	Model	*P_egger_*
TNM stage (III+IV vs. I+II)	4	338	2.93 (1.80-4.77)	<0.001	70.00%	0.018	Random	0.282
Tumor invasion	5	488	2.90 (1.90-4.41)	<0.001	75.90%	0.002	Random	0.469
Lymph node metastasis (positive vs. negative)	5	488	2.99 (1.97-4.54)	<0.001	28.30%	0.233	Fixed	0.918
Distant metastasis (presence vs. absence)	3	292	3.11 (1.68-5.75)	<0.001	92.80%	<0.001	Random	0.509
Histologic grade (poor vs. well, moderate)	3	288	1.00 (0.46-2.20)	0.997	0.00%	0.864	Fixed	0.084

### 3.4 Association of MALAT1 with survival outcomes

**Figure 4:**
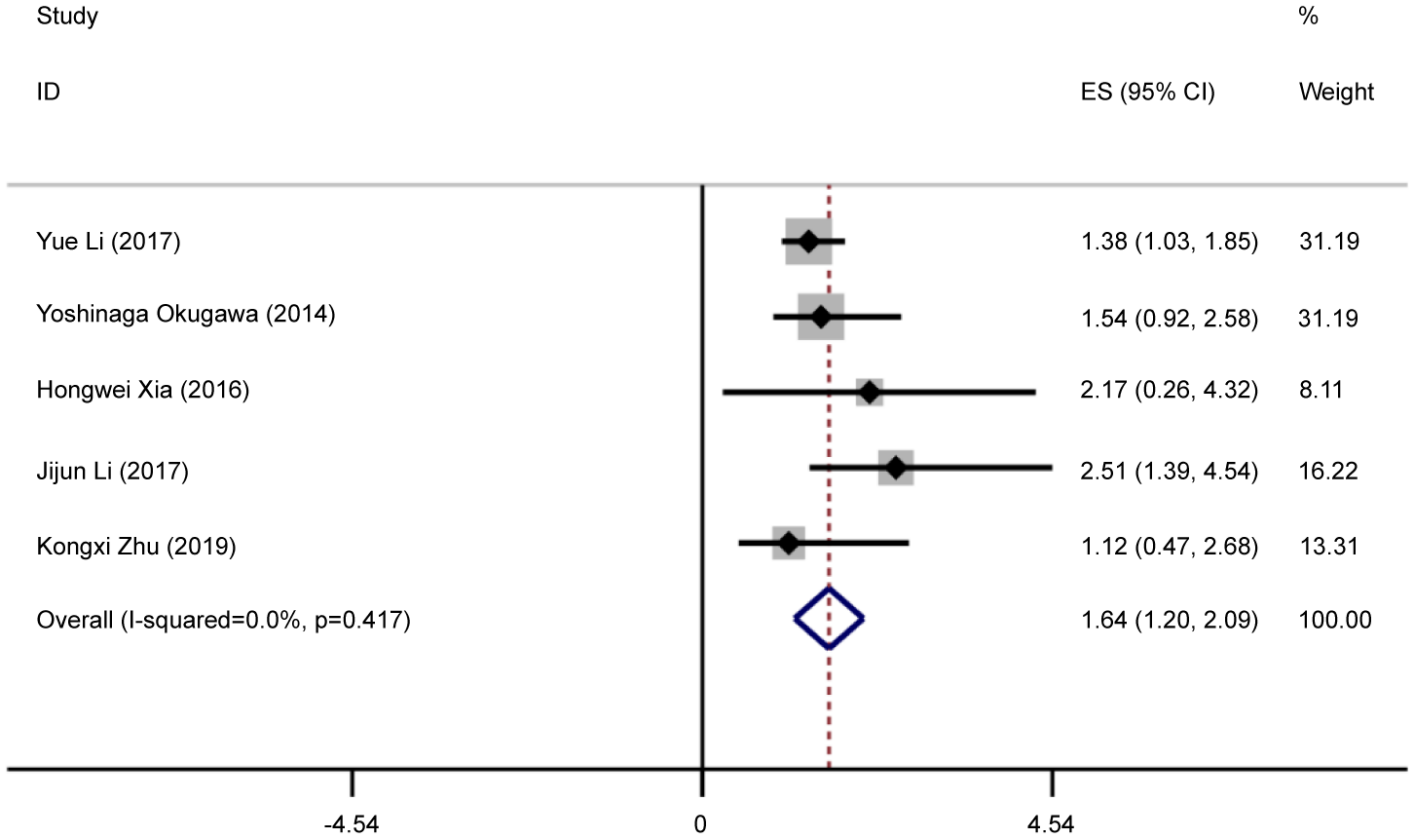
Forest plots of pooled HRs of OS. Elevated MALAT1 expression predicted worse OS in patients with GC (HR = 1.64, 95% CI = 1.20–2.09, P < 0.001).

**Figure 5:**
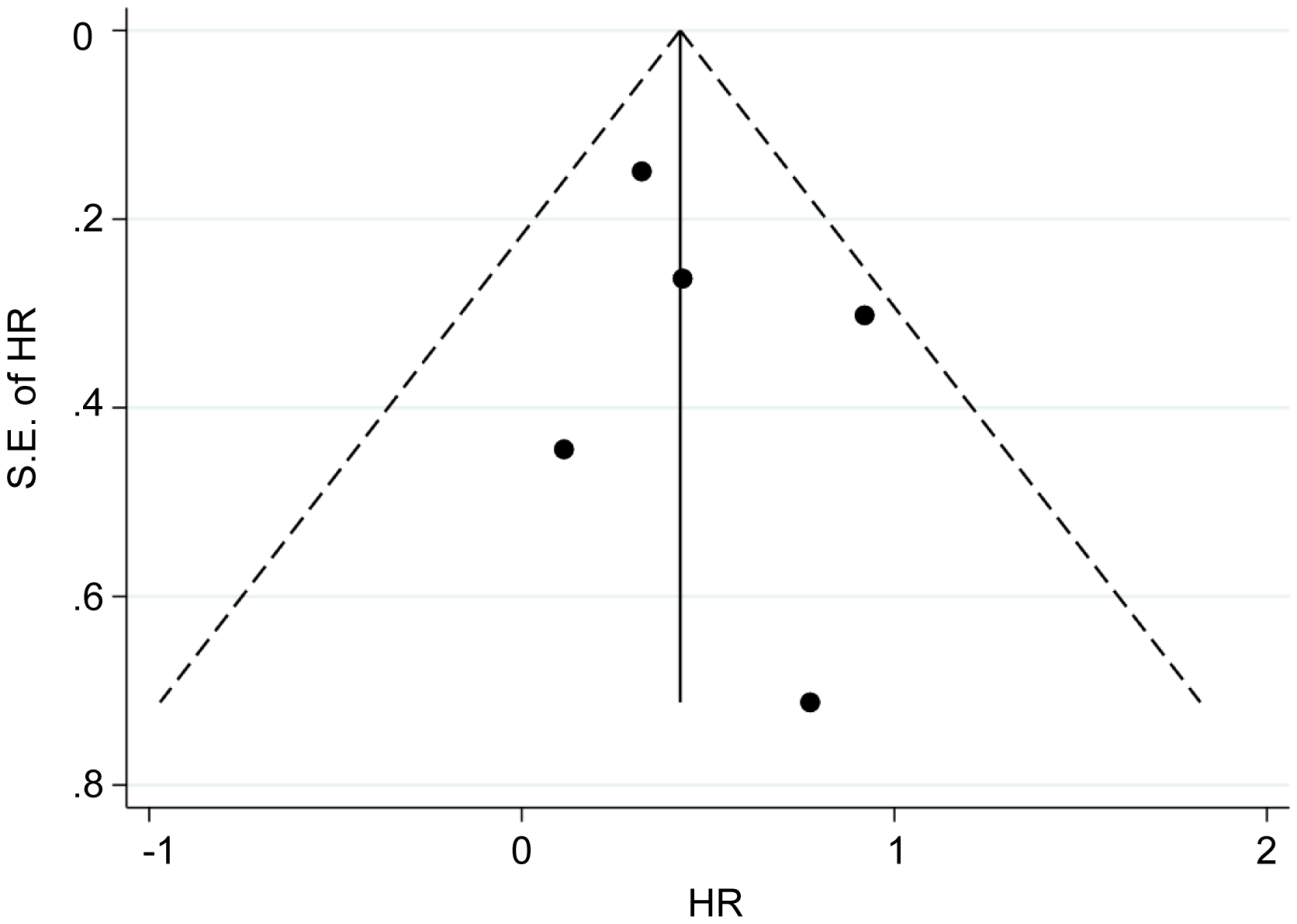
Funnel plots of OS. Funnel plots were used to assess publication bias for OS. The shape of the funnel plots indicated no evidence of asymmetry.

Five eligible studies reported OS according to MALAT1 expression. Because of the lack of heterogeneity across studies (*I^2^ = *0.0%, P_Q_ = 0.417), pooled results were estimated using the fixed-effects model. As presented in **Table 3** and **Figure 4**, elevated MALAT1 expression predicted worse OS in patients with GC (HR = 1.64, 95% CI = 1.20–2.09, P < 0.001). Funnel plots were used to assess publication bias for OS (**Figure 5**), and the shape of the funnel plots indicated no evidence of asymmetry. In addition, Egger’s results revealed no apparent publication bias for OS (P = 0.70).

**Table 3:** Association of MALAT1 expression with survival outcomes.

Author	Year	Samples (n)	MALAT1 high expression (n)	MALAT1 low expression (n)	Data Source	OS result
Yue Li (25)	2017	150	105	45	Direct	1.38(1.03-1.85)
Yoshinaga Okugawa (21)	2014	150	88	62	Direct	1.54(0.92-2.58)
Hongwei Xia (32)	2016	39	NA	NA	Direct	2.169(0.265-4.324)
Jijun Li (27)	2017	78	40	38	Survival Curve Graph	2.51(1.39-4.54)
Kongxi Zhu (29)	2019	64	32	32	Survival Curve Graph	1.12(0.47-2.68)

NA, Not Available.

## 4 Discussion

Despite advances in life and medical sciences, GC remains a global public health problem (37). Therefore, it is necessary to discover the molecular mechanisms of GC progression to prevent tumorigenesis and improve survival. There is increasing evidence that abnormally expressed lncRNAs are involved in progression and tumorigenesis in GC. These lncRNAs participate in many cellular signaling pathways and function as oncogenes or tumor suppressors (38–40). Previous research illustrated that lncRNAs, including MALAT1, are valid predictors of survival outcomes (41, 42).

Several biological roles and abnormal MALAT1 expression have been linked to several cancer types (43–45). In HepG2 cells, MALAT1 inhibition reduced the expression of the transcription factor Oct4, suggesting that MALAT1 can promote stem-like properties in hepatoma cells (46). However, the impact of MALAT1 on GC progression and prognostic outcomes remains controversial. Therefore, to evaluate the clinical and prognostic relevance of MALAT1 in GC, we examined recently published research by meta-analysis.

First, eligible studies were pooled to conduct the meta-analysis. The true link between MALAT1 expression and GC might be more clearly revealed using pooled results because of the correction of confounders that are implicated in many clinical variables. The results illustrated that elevated MALAT1 expression is an effective predictor of GC prognosis, including tumor invasion (T_3_/T_4_ vs. T_1_/T_2_: OR = 2.90, 95% CI = 1.90- 4.41, P <0.001), lymph node metastasis (pooled odds ratio [OR] = 2.99, 95% confidence interval [CI] = 1.97–4.54, P < 0.001), TNM stage (III/IV vs I/II: OR = 2.93, 95% CI: 1.80-4.77, P < 0.001), and distant metastasis (OR = 3.11, 95% CI = 1.68–5.75, P < 0.001). Furthermore, our study found that patients with high MALAT1 expression had worse OS (HR = 1.64, 95% CI = 1.20–2.09, P < 0.001). Therefore, MALAT1 could be a prognostic biomarker for GC. The findings of this study were similar to those of most prior studies, suggesting that MALAT1 is related to poor prognosis in malignant cancers (47–49). High levels of MALAT1 expression in gastric cancer have been shown to encourage metastasis and development (21). According to a recent clinical investigation, MALAT1 has been linked to colorectal cancer, and patients with stage II/III CRC may have a poorer prognosis if their expression of the gene is high (50). Additionally, it has been demonstrated that MALAT1 is overexpressed in hepatocellular carcinoma, which raises the chance of tumor recurrence following liver transplantation (51).

Nevertheless, because of the limited published data, the study had several limitations. First, the use of different qRT-PCR primer sets could have resulted in heterogeneity across studies. Second, studies might have used different cutoffs for low and high MALAT1 expression. Third, some original studies provided incomplete data. Fourth, confounders such as race can cause significant heterogeneity. Finally, the possibility of a “small study effect” could not be dismissed (52, 53). Thus, larger-scale studies are required to confirm these findings. To our knowledge, this is the first meta-analysis to specifically and systematically evaluate the associations of MALAT1 with lymph node metastasis, distant metastasis, and TNM stage in GC even though some studies partly demonstrated the clinicopathological and prognostic significance of MALAT1 in human cancers (54–57).

In summary, this study suggested that high MALAT1 expression is an adverse biomarker for prognostic outcomes, lymph node metastasis, TNM stage, and distant metastasis in GC. MALAT1 might play a key role in GC tumorigenesis. However, before applying MALAT1 in the treatment and management of GC, additionally high-quality, multi-ethnic, and large-scale studies are required to discover the prognostic value and oncogenic function of MALAT1.

## Data availability statement

The original contributions presented in the study are included in the article/supplementary material. Further inquiries can be directed to the corresponding author.

## Author contributions

SB: Conceptualization, Data curation, Formal analysis, Funding acquisition, Visualization, Writing – original draft, Writing – review & editing. JG: Formal analysis, Visualization, Writing – original draft. HZ: Formal analysis, Visualization, Writing – original draft.
