# Supplementary material for: A meta-analysis of the clinicopathological significance of the lncRNA MALAT1 in human gastric cancer
Source: Front Oncol. 2024 Jan 4;13:1257120. doi: 10.3389/fonc.2023.1257120 (PMC10794718; doi:10.3389/fonc.2023.1257120)
Supplement: Supplementary file 1 [file DataSheet_1.docx]

**Supplementary file**

**The search string for the database**

From the time of inception until April 1, 2023, we searched databases including PubMed, Web of Science, Embase, The Cochrane Library, HowNet, and nature. The search was conducted using the following keyword combinations: ("gastric cancer" or "stomach tumor") and ("long noncoding RNA MALAT1" or "lncRNA MALAT1" or "MALAT1"). The retrieved articles' references were thoroughly examined for further pertinent works.

PubMed, "stomach tumor", "lncRNA MALAT1".

Web of Science, "gastric cancer", "MALAT1".

Embase, "gastric cancer", "long noncoding RNA MALAT1".

The Cochrane Library, "gastric cancer", "lncRNA MALAT1".

HowNet, "stomach tumor", "long noncoding RNA MALAT1".

nature, "gastric cancer", "long noncoding RNA MALAT1".
